# Efficacy and safety of rituximab for primary membranous nephropathy with different clinical presentations: a retrospective study

**DOI:** 10.3389/fimmu.2023.1156470

**Published:** 2023-04-28

**Authors:** Shasha Zhang, Jing Huang, Jianwei Dong, Zhuo Li, Mengyao Sun, Yujiao Sun, Bing Chen

**Affiliations:** ^1^ Department of Nephrology, Shandong Provincial Hospital Affiliated to Shandong First Medical University, Jinan, Shandong, China; ^2^ Department of Nephrology, Jinan Shizhong People’s Hospital, Jinan, China; ^3^ Department of Thoracic Surgery, The People’s Hospital of Rongcheng, Rongcheng, Shandong, China

**Keywords:** primary membranous nephropathy, rituximab, clinical remission rate, anti-PLA2R antibody, safety

## Abstract

**Background:**

Rituximab (RTX) is gaining increasing clinical acceptance in the treatment of primary membranous nephropathy (PMN), with demonstrated efficacy and safety. However, there are few clinical studies on RTX for PMN in Asian populations, especially in China.

**Methods:**

To observe and analyse the efficacy and safety of RTX treatment, 81 patients with PMN suffering from nephrotic syndrome (NS) were enrolled and divided into an initial therapy group, a conventional immunosuppressive therapy relapse group, and a conventional immunosuppressive therapy ineffective group according to their pre-RTX treatment background. Patients in each group were followed up for 12 months. The primary outcome was clinical remission at 12 months, and the secondary outcomes were safety and the occurrence of adverse events.

**Results:**

At 12 months, 65 of 81 (80.2%) patients achieved complete (n=21, 25.9%) or partial (n=44, 54.3%) remission after rituximab treatment. Thirty-two of 36 (88.9%) patients in the initial therapy group, 11 of 12 (91.7%) patients in the relapse group and 22 of 33 (66.7%) patients in the ineffective group achieved clinical remission. All 59 patients with positive anti-PLA2R antibodies showed a decreasing trend in antibody levels after RTX treatment, and 55 (93.2%) of them achieved antibody clearance (<20 U/mL). Logistic regression analysis showed that a high anti-PLA2R antibody titer (OR=0.993, P=0.032) was an independent risk factor for nonremission. Adverse events occurred in 18 (22.2%) patients, of which 5 (6.2%) were serious adverse events, and none were malignant or otherwise fatal.

**Conclusion:**

RTX alone can effectively induce remission PMN and maintain stable renal function. It is recommended as the first choice of treatment and is also effective in patients who relapse and have poor responses to conventional immunosuppressive therapy. Anti-PLA2R antibodies can be used as a marker for RTX treatment monitoring, and antibody clearance is necessary to achieve and improve the rates of clinical remission.

## 1 Introduction

Primary membranous nephropathy (PMN) is one of the most common pathological types of adult nephrotic syndrome (NS), in which subepithelial immune complex deposition (mainly IgG and C3) and complement activation are responsible for impaired podocyte function. The course of the disease is highly variable, ranging from spontaneous remission to persistent proteinuria or end-stage kidney disease (ESKD) (1, 2). Spontaneous partial remission occurs in approximately 1/3 of patients (3); however, remission is unlikely to occur in intermediate- and high-risk patients (4, 5). Approximately 40%-50% of patients with untreated persistent NS will develop ESKD and therefore require prompt clinical intervention and treatment.

The Kidney Disease: Improving Global Outcomes (KDIGO) 2012 Clinical Practice Guideline for the Management of Glomerular Diseases recommended glucocorticoids combined with alkylating agents [cyclophosphamide (CTX) or azelaic acid phenylbutyrate] for immunosuppressive therapy (6), but in clinical practice, alkylating agents have demonstrated significant toxic side effects, including myelosuppression, infection, gonadal suppression and an increased risk of tumour formation (7). Other preferred treatment options include calcineurin phosphatase inhibitors (CNIs), such as cyclosporine A or tacrolimus (8). However, recurrence rates of up to 40%-50% have been commonly reported for patients treated with this regimen (9), and patients may become treatment dependent; the risk of chronic nephrotoxicity should not be underestimated. Better alternatives (lower recurrence rate and higher safety) to conventional treatment regimens are urgently needed for PMN and should be further explored and pursued.

Approximately 70-80% of PMN cases are mediated by autoantibodies against the M-type phospholipase A2 receptor (PLA2R) of glomerular podocytes, and an additional 3%-5% of cases are mediated by antibodies against thrombospondin type- 1 domain-containing 7A (THSD7A) (10–12). The pathogenesis of PMN mainly involves T cells secreting various cytokines, such as interleukins, that stimulate B-cell proliferation and activation, and effector B cells secreting specific autoantibodies that bind to PLA2R and THSD7A on the surface of pedunculated cells, forming immune complexes that deposit under the glomerular epithelium, damaging the filtration barrier and triggering proteinuria (13). These significant breakthroughs in the understanding of the disease suggest that PMN is an autoimmune disease that is targeted by podocytes. The pathogenic role of autoantibody-producing B cells in PMN is gradually being understood, providing powerful evidence for B-cell-targeted therapy for the PMN. Rituximab (RTX) is a selective B-cell depleting agent that depletes CD20-positive B cells by specifically binding to the B-cell surface antigen CD20 (14), reducing circulating antibody production and thereby preventing the formation of subepithelial immune deposits in the glomerulus and attenuating glomerular filtration barrier damage, thus leading to PMN remission (15).

Since 2002, various clinical studies, represented by the MENTOR study (16), have confirmed the clinical efficacy and safety of RTX through comparative tracking of RTX and conventional regimens for the treatment of PMN, and the 2021 KDIGO guidelines (5) have also recommended the clinical use of RTX. However, its treatment has been less studied in Asian populations, especially in Chinese populations. This study selected domestic rituximab injection, manufactured by Shanghai Fosun Pharmaceutical Company in China, as the primary investigational agent. This anti-CD20 monoclonal antibody has been studied equivalently with imported rituximab from Roche Diagnostics Gmbh (17, 18), confirming that there is no significant difference in the efficacy of these two products in the treatment of lymphoma. However, there is still a lack of adequate research support for the therapeutic efficacy of this product in membranous nephropathy, especially in the Chinese population. The study was performed to further evaluate the efficacy and safety of RTX in PMN by retrospectively analysing the outcomes of this product when applied alone to PMN in three different treatment settings.

## 2 Materials and methods

### 2.1 Study population

Ninety-eight adult (>18 years old) patients treated at the Department of Nephrology at Shandong Provincial Hospital between 04/2020 and 12/2021 were selected. Seven patients with irregular doses of RTX and 10 patients with less than 12 months of follow-up were excluded; 81 PMN patients were finally enrolled in the retrospective study. Inclusion criteria: (1) IMN diagnosed by renal biopsy. (2) RTX alone chosen as the initial or alternative treatment. (3) Clinical manifestations of NS with proteinuria >3.5 g/24 h and serum albumin <30 g/L prior to RTX treatment. (4) Patients have a well-documented clinical and laboratory examination and have been assessed for potential malignancies, reviewed for pathogenic drugs, screened for hepatitis B/C virus, HIV, autoimmune diseases, etc. by history, physical examination and laboratory tests (serology, imaging, etc.) to exclude factors that may contribute to secondary membranous nephropathy.

The patients were divided into three groups according to their treatment background prior to RTX treatment. The first group was the initial therapy group, in which patients were not given any immunosuppressive therapy before receiving RTX. However, all had received symptomatic supportive treatment such as blood pressure control, reduction of urinary protein levels, angiotensin converting enzyme inhibitor or angiotensin receptor blocker therapy for ≥3 months and remained in persistent NS status. During this treatment, the blood pressure was maintained below 140/90 mmHg, the glomerular filtration rate (eGFR) ≥ 40 ml/min/1.73 m^2^, or the 24-hour endogenous creatinine clearance > 40 ml/min/1.73 m^2^. The second group was the conventional immunosuppressive therapy relapse group (relapse group), in which patients had achieved complete response (CR) or partial response (PR) after conventional glucocorticoid combined with immunosuppressive (CTX or CNI) regimens and had reoccurrence of proteinuria >3.5 g/24 h during drug reduction. The third group was the conventional immunosuppressive therapy ineffective group (ineffective group), in which patients for whom conventional glucocorticoid combined with immunosuppressive (CTX or CNI) regimens were ineffective (not achieving CR or PR) and who received RTX after discontinuation were enrolled. In this group of patients, one subgroup of patients remained in persistent NS status after ≥6 months of treatment with conventional induction remission regimens, and then suspended of immunosuppressive drugs already in use and administration of RTX. The other subgroup of patients achieved CR or PR after receiving conventional induction remission regimens, these patients later relapsed with NS and were given conventional induction remission therapy again for ≥6 months without achieving CR or PR, and then suspended of immunosuppressive drugs already in use and administration of RTX.

This study was carried out in accordance with the Helsinki Declaration, and the study protocol was approved by the Ethics Review Committee of Shandong Provincial Hospital in China (JNKJ: NO. 2020-3028).

### 2.2 Treatment options and follow-up

There were two dosing regimens in this study. In the first, RTX was administered intravenously at 375 mg/m^2^ once a week for 4 weeks as a course of treatment. In the second dosing regimen, RTX was administered intravenously at 1 g/dose used at 2-week intervals for a total of 2 doses as a course of treatment. The 2021 KDIGO guidelines recommend both treatment regimens for use in patients with PMN (5). Previous studies have demonstrated no significant difference in the proportion of CR or PR using the two regimens (19, 20). RTX was dissolved in 9% saline to a concentration of 1 mg/mL and infused at an initial rate of 40 mL/h, which was then gradually increased to 200 mL/h according to the tolerance of each patient. To reduce the infusion response to RTX, patients received methylprednisolone 40 mg, dexamethasone 5 mg, and isoproterenol 25 mg prior to injection.

Follow-up was performed every 3 months, i.e., before RTX treatment and at months 3, 6, 9, and 12 after treatment for study follow-up. Each monitoring index included routine blood, routine urine, liver and kidney function, blood lipid glucose, 24-h urine protein, anti-PLA2R antibody level, and circulating B-cell quantity. We used a standard commercial ELISA (Euroimmune, Lubeck, Germany) to determine anti-PLA2R antibody titers, which were defined as antibody positive when the titer was >20 U/mL. CD19+ B lymphocyte depletion was defined as a concentration of <5 cells/mL. Adverse events associated with rituximab were documented during drug infusion and throughout the follow-up period. Subsequent follow-ups were conducted at 6-month intervals to record the patients’ remission and recurrence.

For both dosing regimens, the decision to reinject with 375 mg/m^2^ × 1-2doses or 1 g × 1-2doses was made at 6 months, depended on the extent of B-cell rebound, anti-PLA2R antibody levels and clinical remission, etc. Subsequent evaluations were repeated every six months or so to see if another injection was needed.

### 2.3 Treatment responses and renal outcomes

The primary outcome was clinical remission at 12 months, and the secondary outcomes were safety and the occurrence of adverse events after medication. To assess treatment response, CR was defined as proteinuria <0.3 g/24h on the premise of stable renal function (eGFR ≥45 ml/min/1.73 m2). PR was defined as proteinuria <3.5 g/24h on the premise of stable renal function (eGFR ≥45 ml/min/1.73 m2), or a decrease in 24-h urinary protein quantification ≥50% from the pretreatment, a ≥30% increase or normalization of serum albumin concentration, and stable or <30% increase in serum creatinine. Patients who did not meet these definitions were considered nonresponders, i.e., they did not achieve clinical remission. Relapse was defined as the reoccurrence of 24-h urine protein quantification >50% of baseline value or >3.5 g in patients who achieved CR or PR. The primary observed endpoint for renal outcomes was a deterioration in renal status or the occurrence of ESKD. Deterioration in renal status was defined as a posttreatment increase in serum creatinine >133 μmol/L or a doubling of baseline serum creatinine levels lasting more than 3 months. ESKD was defined as a creatinine clearance below 15 ml/min at the last follow-up, initiation of dialysis or renal transplantation. A serious adverse event was defined as the occurrence of clinical death or the emergence of a serious pulmonary infection, pulmonary embolism, cerebral infarction, myocardial infarction, or the hospitalization of a patient as a result of an adverse event.

### 2.4 Statistical analysis

Statistical analysis was performed using the statistical software SPSS 22.0. Normally distributed data are described as the means ± SD and were compared by independent t tests or one-way analysis of variance. Nonnormally distributed data are described as the median (interquartile range [IQR]) and were compared by the Mann−Whitney U test or Kruskal−Wallis test. Categorical variables are described as percentages, and Pearson chi-square tests were performed. All probabilities were two-tailed, and the level of significance was set at 0.05. Logistic regression analysis was performed to confirm the potential risk or protective factors for treatment response. The Kaplan−Meier method was used to compare the clinical remission of patients in different background groups after treatment with RTX.

## 3 Results

### 3.1 General baseline parameters

Eighty-one patients with PMN were enrolled with a median age of 53.0 (35.5, 60.0) years, of whom 60 were male and 21 were female. At the time of enrolment, the median protein level was 6.3 (4.4, 10.8) g/24 h, serum albumin was 24.5 ± 5.4 g/L, serum creatinine was 73.6 (62.1, 87.9) μmol/L, and eGFR was 102.3 (74.8, 122.4) mL/min/1.73 m^2^. All 81 patients were tested for anti-PLA2R antibodies in blood; the median level of anti-PLA2R antibodies was 61.0 (17.5, 147.8) U/ml, and 59 patients (72.8%) were tested positive for antibodies (>20 U/mL) (**Table 1**).

**Table 1:** Baseline characteristics of patients with PMN included in this study.

Characteristic	Total(n=81)	Initial therapy (n=36)	Relapse (n=12)	Ineffective(n=33)	P
Male sex, n (%)	60(74.1)	25(69.4)	9(75.0)	26(78.8)	0.674
Age (years)	53.0(35.5, 60.0)	53.0(34.5, 63.0)	55.0(38.0, 60.8)	53.0(36.5, 56.0)	0.922
Urine RBC/μl	20.2(8.8, 38.5)	20.2(9.5, 35.4)	16.5(5.1, 29.4)	25.1(8.9, 66.1)	0.365
Proteinuria (g/24 h)	6.3(4.4, 10.8)	7.1(5.3, 10.9)	5.6(4.3, 7.4)	6.2(4.0, 12.3)	0.348
WBC (×10^9^/L)	7.3(6.2, 9.9)	7.1(6.0, 8.7)	6.9(5.1, 9.7)	8.3(6.8, 11.2)	0.115
Hemoglobin (g/L)	133.0(112.0, 145.0)	135.0(126.5, 145.0)	138.5(121.0, 152.0)	115.0(102.0, 137.0)	**0.001**
Platelet (×10^9^/L)	262.3 ± 62.2	261.4 ± 56.8	258.4 ± 63.3	264.6 ± 69.3	0.953
AST (u/L)	20.5(17.0, 28.0)	22.0(17.0, 28.0)	21.5(17.0, 27.0)	19.0(17.0, 29.0)	0.537
ALT (u/L)	20.0914.0, 25.23)	20.0(15.0, 28.0)	20.5(15.3, 26.3)	19.0(13.0, 25.0)	0.654
Total protein (g/L)	47.9(42.0, 53.2)	48.0(42.2, 53.8)	52.5(46.3, 57.4)	45.1(39.9, 53.3)	0.513
Albumin (g/L)	24.5 ± 5.4	23.9 ± 5.6	27.9 ± 5.6	23.9 ± 4.9	0.062
Globulin (g/L)	23.2 ± 3.5	24.0 ± 3.2	24.2 ± 3.3	22.0 ± 3.6	**0.040**
BUN (mmol/L)	6.5(5.0, 9.4)	6.0(5.0, 9.5)	5.6(4.9, 7.9)	8.0(6.1, 10.5)	0.484
Serum creatinine(μmol/L)	73.6(62.1, 87.9)	69.3(56.2, 81.4)	70.6(63.0, 104.4)	78.9(65.0, 103.1)	0.218
eGFR (mL/min/1.73 m^2^) ^a^	102.3(74.8, 122.4)	107.0(88.9, 124.5)	109.5(66.5, 127.7)	93.0(62.5, 120.3)	0.156
GFR>60, n (%)	71(87.7)	34(94.4)	10(83.3)	27(81.8)	0.249
GFR 40–60, n (%)	10(12.3)	2(5.6)	2(16.7)	6(18.2)	
Cholesterol (mmol/L)	7.1(5.6, 9.2)	8.2(5.9, 9.7)	6.8(5.0, 9.3)	6.8(5.6, 8.8)	0.374
Anti-PLA2R antibody positivity, n (%) ^b^	59(72.8)	26(72.2)	7(58.3)	26(78.8)	0.392
Anti-PLA2R antibodies( >150U/mL)	20(24.7)	9(25.0)	2(16.7)	9(27.3)	0.765
Anti-PLA2R antibodies (U/mL)	61.0(17.5, 147.8)	79.3(18.1, 149.3)	29.2(8.6, 82.9)	70.8(24.0, 182.5)	0.451
Absolute values of CD19(/μl)	215.7(120.2, 334.2)	320.5(198.0, 587.4)	211.7(143.2, 379.5)	156.0(103.8, 279.2)	**0.022**
low-risk, n (%)	3(3.7)	3(3.7)	0	0	
intermediate-risk, n (%)	50(61.7)	21(25.9)	9(11.1)	20(24.7)	0.580
high-risk, n (%)	28(34.6)	12(14.8)	3(3.7)	13(16.0)	0.654

The hemoglobin level in the ineffective group was lower than that in the initial treatment group (P<0.001) and the relapse group (P=0.016). Patients in the initial treatment group had higher globulin levels (P=0.021) and absolute values of CD19 (P=0.007) than those in the ineffective group.

Values are presented as numbers (%), medians (interquartile range), or means ± SD.

RBC, red blood cell; WBC, white blood cell; PMN, primary membranous nephropathy; AST, glutamic oxaloacetic transaminase; ALT, glutamic pyruvic transaminase; BUN, blood urea nitrogen; eGFR, estimated glomerular filtration rate; PLA2R, phospholipase A2 receptor.

a. eGFR was calculated according to the Chronic Kidney Disease Epidemiology Collaboration equation.

b. Anti-PLA2R positivity was defined by a value >20 U/ml.

Values in bold represent P<0.05.

**Figure 1:**
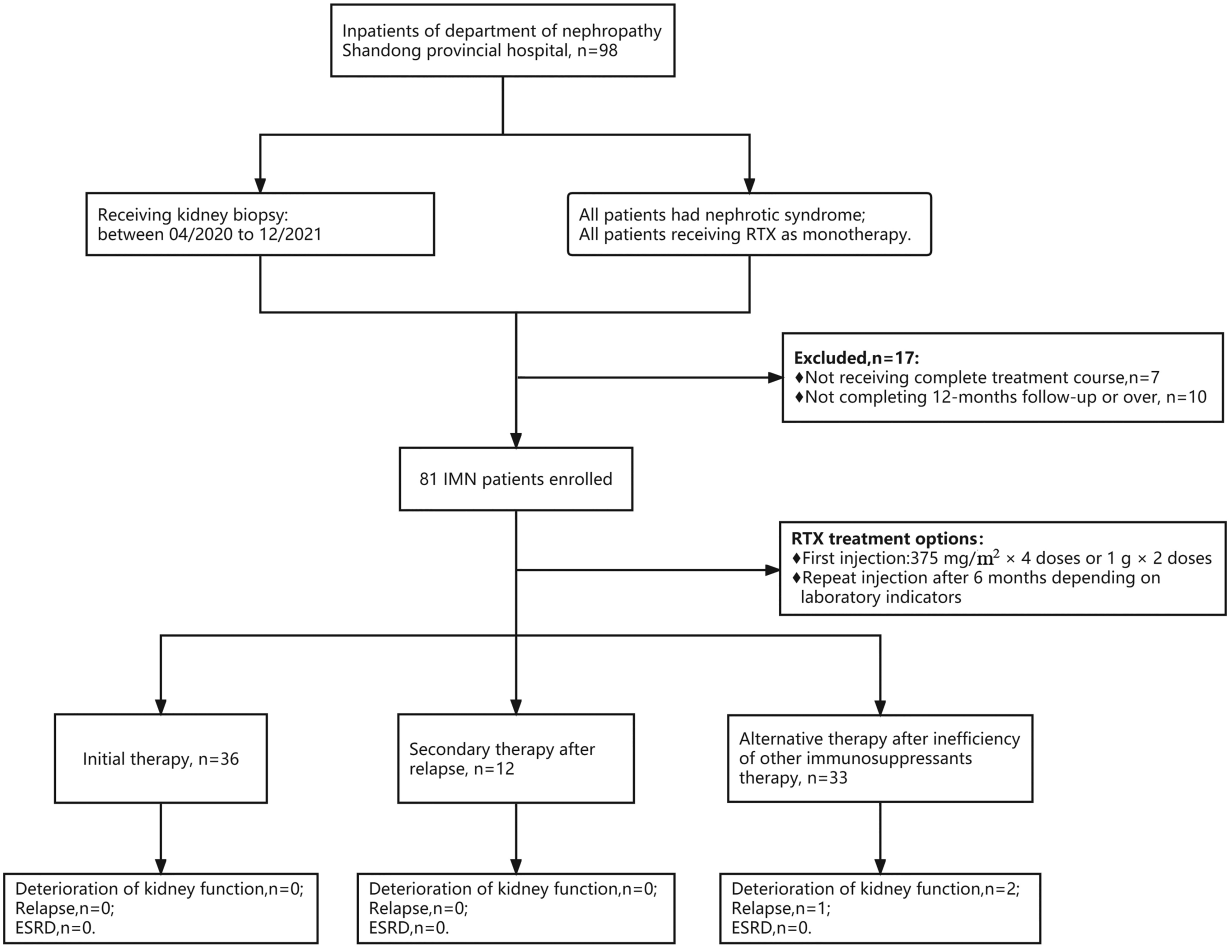
Flow chart of selection for patients with PMN receiving rituximab therapy. A total of 81 PMN patients were enrolled, with 36 patients receiving rituximab as the initial therapy, 12 receiving RTX as a secondary therapy after relapse, and 33 receiving RTX as an alternative therapy after failure of other immunosuppressant therapy. During follow-up, 2 patients had worsening renal function, and 1 patient relapsed, both from the ineffective group. No patients entered ESKD.

Thirty-six patients were enrolled in the initial therapy group, 12 patients were enrolled in the relapse group, and 33 patients were enrolled in the ineffective group (**Figure 1**). Patients in the ineffective treatment group, compared with those in the initial therapy and relapse groups, had low levels of hemoglobin [115.0 (102.0, 137.0) vs. 135.0 (126.5, 145.0) vs. 138.5 (121.0, 152.0) g/L, P=0.001], globulin [22.0 ± 3.6 vs. 24.0 ± 3.2 vs. 24.2 ± 3.3 g/L, P=0.040] and absolute CD19 values [156.0 (103.8, 279.2) vs. 320.5 (198.0, 587.4) vs. 211.7 (143.2, 379.5)/μl, P=0.022], and had high proportion of eGFR <60 mL/min/1.73 m^2^ [6/33 (18.2%) vs. 2/36 (5.6%) vs. 2/12 (16.7%), P=0.249, difference not statistically significant] (**Table 1**). The eGFR was >60 mL/min/1.73 m^2^ in 71 patients and 40-60 mL/min/1.73 m^2^ in 10 patients. The clinical remission rate was higher in patients with high levels of eGFR than in those with low levels (83.1% vs. 60.0%, P=0.086, difference not statistically significant). There were 3 low-risk patients, 50 intermediate-risk patients and 28 high-risk patients. Fifty-seven (70.4%) patients received repeat RTX injections during follow-up after completion of full-dose (375 mg/m^2^×4 doses or 1 g×2 doses) RTX treatment regimens.

### 3.2 Efficacy assessment

All patients completed at least 12 months of follow-up. At 12 months, 65 of 81 (80.2%) patients achieved CR (n=21, 25.9%) or PR (n=44, 54.3%) with rituximab treatment. Thirty-two of 36 (88.9%) patients in the initial therapy group achieved clinical remission, of whom 12 (33.3%) patients achieved CR and 20 (55.6%) patients achieved PR. Eleven of 12 (91.7%) patients in the conventional immunotherapy relapse group achieved clinical remission, of whom 3 (25.0%) patients achieved CR and 8 (66.8%) patients achieved PR. Twenty-two of 33 (66.7%) patients in the conventional immunotherapy ineffective group achieved clinical remission, of whom 6 (18.2%) patients achieved CR and 16 (48.5%) patients achieved PR (**Table 2**).

**Table 2:** Complete remission or composite (complete or partial remission) at 3–12 months by intention-to-treat analysis.

Study Time Points	No. of Patients with Remission/Total No. (%)				P
	Total	Initial therapy	Relapse	Ineffective	
Complete remission					
3 months	1/81(1.2)	1/36(2.7)	0(0)	0(0)	0.531
6 months	5/81(6.2)	3/36(8.3)	1/12(8.3)	1/33(3.0)	0.622
9 months	12/81(14.8)	7/36(19.4)	3/12(25.0)	2/33(6.1)	0.165
12 months	21/81(25.9)	12/36(33.3)	3/12(25.0)	6/33(18.2)	0.365
Complete or partial remission					
3 months	31/81(32.3)	17/36(47.2)	6/12(50.0)	8/33(24.4)	0.097
6 months	39/81(48.1)	21/36(58.3)	7/12(58.3)	11/33(33.3)	0.086
9 months	52/81(64.2)	26/36(72.2)	10/12(83.3)	16/33(48.5)	**0.039**
12 months	65/81(80.2)	32/36(88.9)	11/12(91.7)	22/33(66.7)	**0.038**

The primary outcome was complete remission at 12 months. The overall remission rate was 80.2% (65/81). The initial therapy group had a higher remission rate than the ineffective group at 9 months (72.2%vs.48.5%, P=0.044) and at 12 months (88.9% vs. 66.7%, P=0.025). The relapse group had a higher remission rate than the ineffective group at 12 months (91.7%vs.66.7%, P=0.036).

Values in bold represent P<0.05.

**Figure 2:**
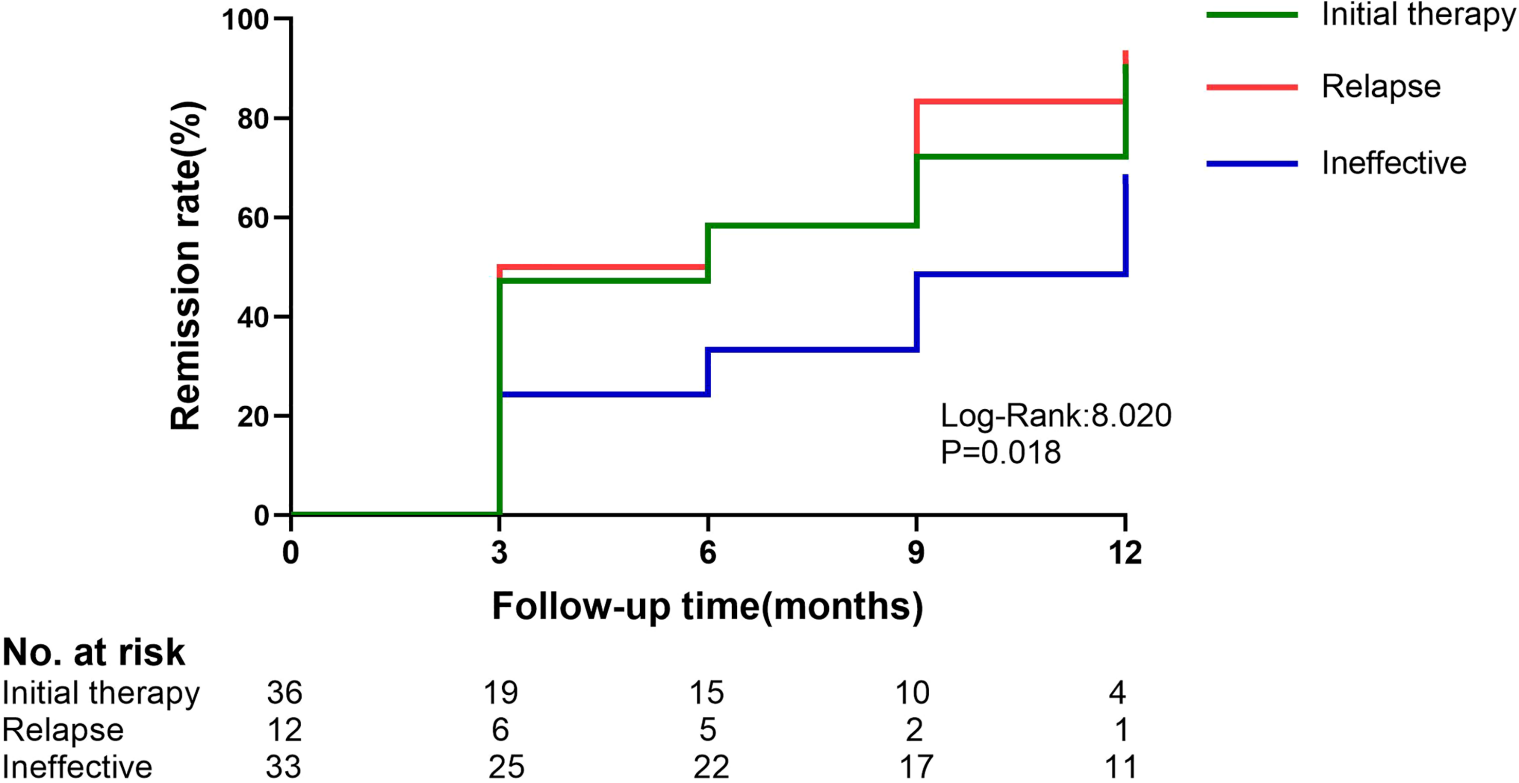
Kaplan–Meier estimates of the composite remission (complete or partial) in the initial therapy,relapse and ineffective group. The initial therapy group and the ineffective group have a P-value of 0.013.

During the follow-up period, the clinical remission rate of the patients gradually increased with longer follow-up. The ineffective group had a lower remission rate than the initial therapy group (66.7% vs. 88.9%, P=0.025). The difference in clinical remission rates between the two groups was already significant at 3 months (P=0.047) and was maintained throughout the study period (**Figure 2**).

### 3.3 Changes in laboratory indexes

**Figure 3:**
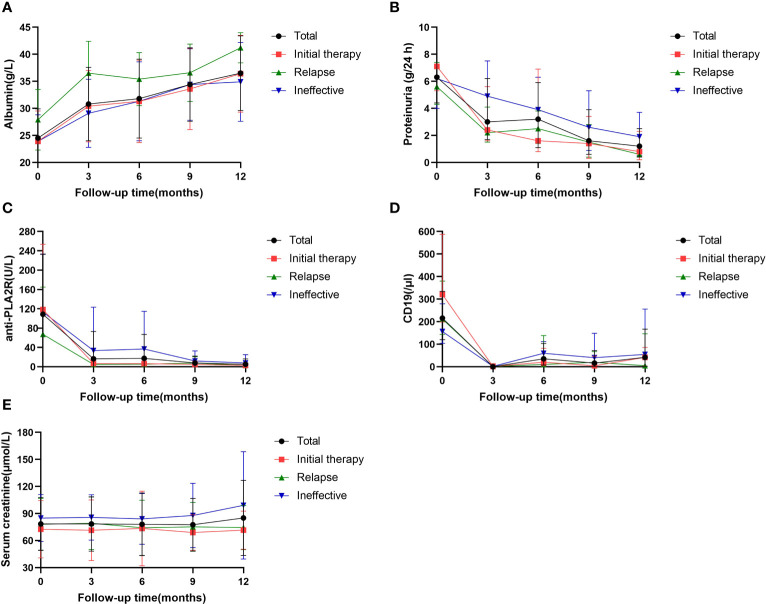
Serial levels of albumin **(A)**, proteinuria **(B)**, anti-PLA2R antibody **(C)**, the absolute values of CD19 **(D)** ana serum creatinine **(A)** after rituximab treatment in patients who had been followed up for 12 months. Data are presented as the medians (interquartile range) over time **(B, D)** or mean ± SD **(A, C, E)**.

During the 12-month monitoring period, all PMN patients showed an overall decreasing trend in anti-PLA2R antibodies, 24-h urine protein quantification, and absolute CD19 values and an upward trend in serum albumin (**Figure 3**). The results at month 12 showed a decrease in anti-PLA2R antibodies from 61.0 (17.5, 147.8) U/mL to 2.0 (2.0, 3.7) U/mL in all PMN patients, with overall antibody levels becoming negative. The 24-hour urine protein levels dropped from 6.3 (4.4, 10.8) g/24 h to 1.2 (0.4, 2.5) g/24 h. Serum albumin gradually increased from 24.5 ± 5.4 g/L to 38.0 (33.0, 40.7) g/L, and the difference between the three groups was statistically significant (P=0.033). The relapse group had a higher increase in albumin level than the initial therapy group (P=0.047) and the ineffective group (P=0.009) (**Table 3**). One of the 68 (1.5%) patients relapsed after achieving clinical remission, and two patients developed worsening renal function, both in the ineffective group. No patients progressed to ESKD.

**Table 3:** Clinical characteristics of PMN patients after rituximab treatment for 12 months.

	Total	Initial therapy	Relapse	Ineffective	P
Time to reach remission (months)	6.6 ± 3.7	6.0 ± 3.7	6.0 ± 3.5	7.7 ± 3.8	0.127
Proteinuria (g/24 h)	1.2(0.4, 2.5)	0.8(0.2, 2.3)	0.6(0.2, 0.9)	1.9(0.5, 3.7)	0.224
Albumin (g/L)	38.0(33.0, 40.7)	38.8(33.7, 40.9)	41.0(39.0, 43.0)	36.6(29.4, 38.4)	**0.033**
Anti-PLA2R antibody positivity, n(%)	4(4.9)	1(2.8)	0(0)	3(9.1)	0.334
Anti-PLA2R antibodies (U/mL)	2.0(2.0, 2.9)	2.0(2.0, 2.0)	2.0(2.0, 3.6)	2.0(2.0, 5.7)	0.143
Serum creatinine (μmol/L)	78.4(62.1, 90.3)	72.3(53.0, 81.4)	71.5(60.0, 91.1)	85.9(74.9, 101.0)	0.072
Absolute values of CD19 (/μl)	41.7(1.8, 166.5)	41.7(2.8, 85.9)	4.7(0.6, 146.4)	54.5(2.2, 255.6)	0.230

The serum albumin level in the relapse group was higher than that in the initial treatment group (P=0.047) and the ineffective group (P=0.009).

PLA2R, phospholipase A2 receptor.

Values in bold represent P<0.05.

All 59 patients with positive anti-PLA2R antibodies showed a decrease in antibody levels after RTX treatment, and 55 patients (93.2%) achieved antibody conversion to antibody negativity (<20 U/mL), including 10 with levels <4 U/ml and 35 with levels <2 U/ml. Forty-four of 55 (80.0%) patients achieved clinical remission. Among the 4 patients with nonconverted antibody negativity (all B-cell levels <5/mL), 1 patient (25.0%) achieved clinical remission. The remission rates were significantly different between antibody conversion and nonconversion (P=0.013). Of the 81 patients, the patients with high antibody titers (>150 U/mL) had significantly lower clinical remission rates than patients with low titers (50% vs. 90.16%, P < 0.001). Seventy-one (87.7%) patients were positive for histological anti-PLA2R antibodies, and their clinical remission rate was 78.9% (15/71); the remission rate in antibody-negative patients was 90% (9/10) (P=0.408, no statistically significant difference).

### 3.4 Analysis of risk factors

Compared with patients who achieved clinical remission, nonresponders had higher levels of anti-PLA2R antibodies [204.5 (39.5, 331.4) vs. 47.5 (11.5, 104.7) U/mL, P=0.010], total protein [42.9 (38.4, 51.2) vs. 48.6 (43.0, 54.2) g/L, P=0.019], globulin [21.2 ± 2.8 vs. 23.7 ± 3.5 g/L, P=0.012], and C3 [1.1 (1.0, 1.1) vs. 1.2 (1.1, 1.3) g/L, P=0.035] (**Table 4**). Univariate logistic regression analysis showed that anti-PLA2R antibody titer (OR=0.994, P=0.005), cholesterol (OR=0.807, P=0.040), and blood creatinine (OR=0.979, P=0.033) were risk factors for nonremission, whereas total protein (OR=1.104, P=0.026) and globulin (OR= 1.256, P=0.017) were protective factors, and a high anti-PLA2R antibody titer (OR=0.993, P=0.032) was an independent risk factor for nonremission (**Table 5**).

**Table 4:** Composite comparisons of clinical features of patients with PMN between responders and nonresponders. n=81.

	Responders ^a^ n=65	Nonresponders n=16	P^d^
Male sex, n (%)	46 (70.8)	14 (87.5)	0.171
Age (years)	53.0 (36.5, 60.0)	51(33.3, 57.3)	0.643
Urine RBC/HPF	19.4(8.6, 34.1)	34.8(9.9, 70.0)	0.129
Proteinuria (g/24 h)	6.2(4.4, 10.4)	7.7(4.6, 12.9)	0.243
Hemoglobin (g/L)	134(113.5, 145.0)	127.0(110.0, 141.0)	0.285
Total protein (g/L)	48.6(43.0, 54.2)	42.9(38.4, 51.2)	**0.019**
Albumin (g/L)	25.0 ± 5.2	22.6 ± 6.2	0.129
Globulin (g/L)	23.7 ± 3.5	21.2 ± 2.8	**0.012**
Serum creatinine (μmol/L)	72.7(59.7, 85.0)	79.0(63.6, 123.5)	0.133
eGFR (mL/min/1.73 m^2^) ^b^	100.2 ± 25.6	93.8 ± 39.2	0.542
Cholesterol (mmol/L)	6.9(5.5, 8.8)	8.4(6.6, 11.2)	0.059
Anti-PLA2R antibody positivity, n (%) ^c^	45(69.2)	14(87.5)	0.141
Anti-PLA2R antibodies (U/mL)	47.5(11.5, 104.7)	204.5(39.5, 331.4)	**0.010**
C3 (g/L)	1.2(1.1, 1.3)	1.1(1.0, 1.1)	**0.035**
Absolute values of CD19 (/μl)	217.5(119.2, 390.3)	210.5(123.5, 326.0)	0.535

eGFR, estimated glomerular filtration rate; PLA2R, phospholipase A2 receptor; C3, complement.

a. Responders are defined as patients who achieve clinical remission.

b. eGFR was calculated according to the Chronic Kidney Disease Epidemiology Collaboration equation.

c. Anti-PLA2R positivity defined by a value >20 RU/ml.

d. Values in bold represent P<0.05.

**Table 5:** Risk factors for nonremission in patients with PMN receiving rituximab therapy (logistic regression).

	Univariate analysis		Multivariate analysis	
	OR (95% CI)	P	OR (95% CI)	P
Male sex	2.891(0.598, 13.968)	0.186		
Age (years)	1.004(0.965, 1.043)	0.854		
Proteinuria (g/24 h)	0.962(0.871, 1.062)	0.446		
ALT	1.086(0.990, 1.192)	0.080		
AST	1.013(0.969, 1.059)	0.563		
Total protein (g/L)	1.104(1.012, 1.204)	**0.026**	0.051(0.834, 1.324)	0.676
Albumin (g/L)	1.088(0.975, 1.215)	0.132		
Globulin (g/L)	1.256(1.041, 1.514)	**0.017**	1.268(0.834, 1.926)	0.266
Cholesterol (mmol/L)	0.807(0.658, 0.990)	**0.040**	0.853(0.603, 1.208)	0.371
Serum creatinine (μmol/L)	0.979(0.959, 0.998)	**0.033**	0.984(0.960, 1.009)	0.203
eGFR (mL/min/1.73 m^2^) ^a^	1.008(0.989, 1.027)	0.425		
Anti-PLA2R antibody positivity, n (%) ^b^	3.111(0.646, 14.991)	0.157		
Anti-PLA2R antibodies (U/mL)	0.994(0.990, 0.998)	**0.005**	0.993(0.986, 0.999)	**0.032**
Absolute values of CD19 (/μl)	1.002(0.999, 1.005)	0.308		

ALT, glutamic pyruvic transaminase; AST, glutamic oxaloacetic transaminase; eGFR, estimated glomerular filtration rate; PLA2R, phospholipase A2 receptor.

a. eGFR was calculated according to the Chronic Kidney Disease Epidemiology Collaboration equation.

b. Anti-PLA2R positivity was defined by a value >20 RU/ml.

c. Values in bold represent P<0.05.

### 3.5 Adverse events

Adverse events occurred in 18 (22.2%) participants during the study, and serious adverse events occurred in 5 (6.2%) patients (2 patients hospitalized for herpes zoster and 3 patients hospitalized for pulmonary infection). The most common adverse reactions were infusion reactions, including rash, erythema, pruritus, runny nose, and irritability, all of which resolved spontaneously after the infusion was completed. Only patients with severe herpes zoster and pulmonary infections received systemic therapy, and all patients made a full recovery (**Table 6**).

**Table 6:** Adverse events in all patients with PMN receiving rituximab.

Events	Patients (n)	No. of events (n)
**Any adverse event**	18	25
**Serious adverse events**	5	6
**Fatal**	0	0
**Nonfatal**	5	6
Fever, pulmonary infection	2	2
Herpes zoster	3	4
**Nonserious adverse events**	13	19
Infusion reactions*	6	9
Swelling of the limb on the infusion side with joint pain	2	3
Diarrhoea	1	2
Ileus	1	1
Nausea and dizziness with transient tinnitus	1	1
Flustered	1	2
Weight loss	1	1

*Infusion reactions include rash, erythema, pruritus, runny nose, and irritability.

## 4 Discussion

The 2021 KDIGO guidelines (5) began recommending RTX for management of PMN, and the efficacy and safety have been confirmed for clinical application. However, its therapeutic effects have been less researched in Asian populations, especially in the Chinese population. This study further confirmed the therapeutic efficacy and safety of RTX in the treatment of Chinese PMN patients in a retrospective analysis, which showed that most patients achieved clinical remission, anti-PLA2R antibody levels decreased significantly or turned negative, renal function remained relatively stable, and no patients progressed to ESKD. The results of this study emphasize the necessity of antibody clearance to achieve and improve clinical remission.

The results showed an overall remission rate of 80.2% at month 12, slightly higher than that in previous studies on the efficacy of RTX (21–26). When rituximab was administered as an initial therapy, the clinical remission rate was 88.9%, which was better than the previous remission rate of 69.1% reported by Ruggenenti et al. (21), the 60% in the MENTOR study (22), the 62% in the RI-CYCLO study (23), and the 64.9% at month 12 in the GEMRITUX study (24). Compared to the initial therapy group, the ineffective group had a lower clinical remission rate, with 66.7% of patients achieving remission, which was better than the remission rate of 50.0% reported by Ruggenenti et al. (25) and 41.7% reported in the Peking University First Hospital study (26). The remarkably greater remission rates in the initial therapy group of the current study compared with those of previous trials may be explained by several reasons. First, the inclusion of patients differed, with the current study enrolling patients with a relatively higher eGFR, relatively more low- and intermediate-risk patients, and relatively fewer high-risk patients. Second, Chinese patients have a smaller body surface area than Western patients, and for patients receiving a 1 g x 2 dose regimen, the same dose of drug is relatively more available in patients with a smaller body surface area, i.e., a longer half-life and longer duration of action. Additionally, the patients in this study all received a standard regimen of full-dose RTX (375 mg/m^2^ × 4 doses or 1 g × 2 doses) at the time of the first course of administration; additionally, most (70.4%) of the patients were given a second course of RTX infusion after 6 months, and full-dose treatment may facilitate a better treatment response (27). In fact, the issue of the optimal RTX dose in PMN therapy remains somewhat controversial, with RTX doses varying widely from a single dose of 375 mg/m^2^ to four doses of 375 mg/m^2^ in different studies, but the mainstream consensus suggests that adequate doses of RTX are more efficacious. There is a lack of randomized controlled cohort studies of low-dose versus full-dose RTX infusion in China and abroad, and further exploration of this issue is warranted (28).

Patients in the ineffective group had a lower overall level of hemoglobin, and a higher proportion of the patients had an eGFR <60 mL/min/1.73 m^2^ than those in the initial therapy and relapse groups, indicating a more severe degree of renal injury. Previous studies have shown that patients with tubulointerstitial lesions and renal impairment respond worse than patients with normal renal function. Additionally, the significantly lower globulin and absolute CD19 values indicated the relatively low overall immunity of the patients in the ineffective group. The above could explain the low clinical remission rate in the ineffective group. The absolute CD19 values were significantly lower in the ineffective and relapse groups than in the initial groups, and we considered that previous immunosuppression had an immunosuppressive effect on the patients, reducing their immunity, although remission of proteinuria was not achieved.

Anti-PLA2R antibodies are key markers of PMN (13, 24, 29) that can be effectively cleared by RTX. The outcomes of this study showed that antibody levels decreased significantly in antibody-positive patients at 12 months after RTX treatment; additionally, patients achieving clinical remission had slightly lower antibody levels than nonresponders prior to RTX treatment, and the clinical remission rates were distinctly different between antibody-depleted and nondepleted patients. Clinical remission rates were significantly lower in patients with high antibody titers (>150 U/mL) than in patients with low titers. These data suggest the effectiveness of RTX in clearing anti-PLA2R antibodies and the importance of antibody elimination in obtaining clinical remission. Remuzzi et al. (4) monitored 132 PMN patients undergoing RTX treatment in terms of their anti-PLA2R antibodies and found that patients with high antibody titers had a lower probability of achieving CR or PR than patients with low titers. Ruggenenti et al. (21) found a correlation between anti-PLA2R antibody levels at baseline and the clinical course of patients on RTX therapy, with antibody titers rising during clinical activity and falling before clinical remission. Therefore, anti-PLA2R antibodies are a valid identifier for monitoring the effect of RTX treatment (22).

Logistic analysis of the elements that may influence the treatment outcome found that anti-PLA2R antibody titer (OR=0.994, P=0.005), cholesterol (OR=0.807, P=0.040) and creatinine (OR=0.979, P=0.033) were risk factors for nonremission, total protein (OR=1.104, P=0.026) and globulin (OR=1.256, P=0.017) were protective factors, and a high anti-PLA2R antibody titer (OR=0.993, P=0.032) was an independent risk factor for nonremission. Brand J et al. (7) similarly proposed that serum total protein levels could be a protective factor in the treatment of PMN. Nonresponders had lower total protein, globulin and albumin levels and higher anti-PLA2R antibody titers, total cholesterol and creatinine levels than patients who achieved clinical remission. These outcomes also indirectly reaffirm that patients with less proteinuria, higher albumin and globulin, and lower anti-PLA2R antibodies, total serum cholesterol, and creatinine (i.e., patients at low to intermediate risk) are more likely to experience remission, suggesting that appropriate threshold migration treatment may benefit more patients and lead to earlier clinical remission.

Regarding adverse events, RTX was well tolerated by most of the patients, with 22.2% of patients experiencing adverse events and only 6.2% experiencing serious adverse events. The rate of adverse events in our study was lower than that of previous relevant studies, which reported values of 50-80% (19), and other studies reported serious adverse event rates of 0-17% (30, 31). This discrepancy may be due to the retrospective nature of this study, which may have resulted in the omission of some minor adverse events. In addition, the most common type of adverse reaction in previous studies was infusion reactions (7), which was relatively infrequent in the present study; this may have been due to the use of anti-allergy medication prior to infusion and the limited rate of infusion to avoid or ameliorate infusion-related events to some extent (32, 33). For serious adverse events, relevant data were carefully verified in this study, and none of them were malignant or fatal. This study provided additional evidence suggesting that RTX is safe for treating PMN.

Cravedi et al. (34) evaluated the costs required for RTX versus glucocorticoids combined with cyclophosphamide treatment for 6 months, and while RTX incurred higher expenses, the latter was associated with more adverse events. By taking the costs of treating adverse events into account, the total costs for glucocorticoids combined with cyclophosphamide may exceed those for RTX. Treatment with cyclosporine A may lead to a total cost of treatment that is greater than the cost of treatment with RTX. Hamilton et al. (35) showed that RTX treatment regimens were relatively inexpensive after 5 years of PMN management; furthermore, RTX treatment expenditures were relatively lower the longer the duration of treatment. Despite the relatively higher cost of single-dose RTX, it remains a cost-effective regimen in the medium- to long-term management of PMN. Thus, rituximab should be recommended as a first-line treatment for patients with PMN, rather than as remedial therapy, from both an efficacy and an economic perspective.

In conclusion, treatment with RTX alone for PMN results in a high clinical remission rate and has a relatively low impact on patients’ renal function. RTX has a high safety profile and is less prone to adverse events. RTX is recommended as the preferred treatment option, and demonstrated efficacy in patients with PMN who have relapsed and are not effectively relieved when treated with conventional immunotherapy. Reasonable and standardized treatment application and regular monitoring of anti-PLA2R antibody levels during therapy can help to improve the effectiveness of treatment and reduce the incidence of adverse events.

## Data availability statement

The raw data supporting the conclusions of this article will be made available by the authors, without undue reservation.

## Ethics statement

The studies involving human participants were reviewed and approved by the Ethics Review Committee of Shandong Provincial Hospital in China (JNKJ: NO. 2020-3028). Written informed consent for participation was not required for this study in accordance with the national legislation and the institutional requirements.

## Author contributions

SZ collected and analyzed the data, authored and reviewed drafts of the paper. JH, JD and MS collected the data, prepared figures and tables. ZL and YS analyzed the data and reviewed drafts of the paper. BC conceived and designed the study, analyzed the data, authored and reviewed drafts of the paper. All authors contributed to the article and approved the submitted version.

## References

[1] Schieppati A Mosconi L Perna A Mecca G Bertani T Garattini S. Prognosis of untreated patients with idiopathic membranous nephropathy. New Engl J Med (1993) 329(2):85–9. doi: 10.1056/NEJM199307083290203 8510707

[2] Glassock R. Diagnosis and natural course of membranous nephropathy. Semin Nephrol (2003) 23(4):324–32. doi: 10.1016/s0270-9295(03)00049-4 12923720

[3] Polanco N Gutierrez E Covarsi A Ariza F Carreno A Vigil A. Spontaneous remission of nephrotic syndrome in idiopathic membranous nephropathy. J Am Soc Nephrol (2010) 21(4):697–704. doi: 10.1681/ASN.2009080861 20110379 PMC2844306

[4] Ruggenenti P Debiec H Ruggiero B Chianca A Pelle T Gaspari F. Anti-phospholipase A2 receptor antibody titer predicts post-rituximab outcome of membranous nephropathy. J Am Soc Nephrol (2015) 26(10):2545–58. doi: 10.1681/ASN.2014070640 PMC4587688 25804280

[5] Kidney Disease: Improving Global Outcomes Glomerular Diseases Work G. Kdigo 2021 clinical practice guideline for the management of glomerular diseases. Kidney Int (2021) 100(4S):S1–S276. doi: 10.1016/j.kint.2021.05.021 34556256

[6] Kidney Disease: Improving Global Outcomes (Kdigo) Glomerulonephritis Work Group. Kdigo clinical practice guideline for glomerulonephritis. Kidney Int Suppl (2012) 2(2):139–274.

[7] van den Brand J Ruggenenti P Chianca A Hofstra JM Perna A Ruggiero B. Safety of rituximab compared with steroids and cyclophosphamide for idiopathic membranous nephropathy. J Am Soc Nephrol (2017) 28(9):2729–37. doi: 10.1681/ASN.2016091022 PMC5576929 28487395

[8] Wu L Lai J Ling Y Weng Y Zhou S Wu S. A review of the current practice of diagnosis and treatment of idiopathic membranous nephropathy in China. Med Sci Monit (2021) 27:e930097. doi: 10.12659/MSM.930097 33550324 PMC7876949

[9] Alfaadhel T Cattran D. Management of membranous nephropathy in Western countries. Kidney Dis (Basel) (2015) 1(2):126–37. doi: 10.1159/000437287 PMC4934807 27536673

[10] Tomas NM Beck LH Jr. Meyer-Schwesinger C Seitz-Polski B Ma H Zahner G. Thrombospondin type-1 domain-containing 7a in idiopathic membranous nephropathy. N Engl J Med (2014) 371(24):2277–87. doi: 10.1056/NEJMoa1409354 PMC4278759 25394321

[11] Beck LH Jr. Bonegio RG Lambeau G Beck DM Powell DW Cummins TD. M-type phospholipase A2 receptor as target antigen in idiopathic membranous nephropathy. N Engl J Med (2009) 361(1):11–21. doi: 10.1056/NEJMoa0810457 19571279 PMC2762083

[12] Allinovi M Lugli G Rossi F Palterer B Almerigogna F Caroti L. Accuracy of serum Pla2r antibody detected by indirect immunofluorescence in diagnosing biopsy-proven primary membranous nephropathy: a single-center experience and a systematic review of the literature. J Nephrol (2023) 36(2):281–3. doi: 10.1007/s40620-022-01528-1 36462140

[13] Ruggenenti P Fervenza FC Remuzzi G. Treatment of membranous nephropathy: time for a paradigm shift. Nat Rev Nephrol (2017) 13(9):563–79. doi: 10.1038/nrneph.2017.92 28669992

[14] Reff KC ME Chambers KS Chinn PC Leonard JE Raab R Newman RA. Depletion of b cells in vivo by a chimeric mouse human monoclonal antibody to Cd20. Blood (1994) 83(2):435–45. doi: 10.1182/blood.V83.2.435.435 7506951

[15] Kattah AG FF. Rituximab: emerging treatment strategies of immune mediated glomerular disease. Expert Rev Clin Immunol (2012) 8(5):413–21. doi: 10.1586/eci.12.26 22882216

[16] Ponticelli C Moroni G. Rituximab or cyclosporine for membranous nephropathy. New Engl J Med (2019) 381(17):1688–9. doi: 10.1056/NEJMc1910393 31644856

[17] Shi Y Zhang Q Han X Qin Y Ke X Su H. Phase 1 studies comparing safety, tolerability, pharmacokinetics and pharmacodynamics of Hlx01 (a rituximab biosimilar) to reference rituximab in Chinese patients with Cd20-positive b-cell lymphoma. Chin J Cancer Res (2021) 33(3):405–16. doi: 10.21147/j.issn.1000-9604.2021.03.11 PMC8286892 34321836

[18] Shi Y Song Y Qin Y Zhang Q Han X Hong X. A phase 3 study of rituximab biosimilar Hlx01 in patients with diffuse Large b-cell lymphoma. J Hematol Oncol (2020) 13(1):38. doi: 10.1186/s13045-020-00871-9 32299513 PMC7164184

[19] Fervenza FC Cosio FG Erickson SB Specks U Herzenberg AM Dillon JJ. Rituximab treatment of idiopathic membranous nephropathy. Kidney Int (2008) 73(1):117–25. doi: 10.1038/sj.ki.5002628 17943078

[20] Ruggenenti P Chiurchiu C Brusegan V Abbate M Perna A Filippi C. Rituximab in idiopathic membranous nephropathy: a one-year prospective study. J Am Soc Nephrol (2003) 14(7):1851–7. doi: 10.1097/01.asn.0000071511.35221.b3 12819245

[21] Ruggenenti P Cravedi P Chianca A Perna A Ruggiero B Gaspari F. Rituximab in idiopathic membranous nephropathy. J Am Soc Nephrol (2012) 23(8):1416–25. doi: 10.1681/ASN.2012020181 PMC3402291 22822077

[22] Fervenza FC Appel GB Barbour SJ Rovin BH Lafayette RA Aslam N. Rituximab or cyclosporine in the treatment of membranous nephropathy. N Engl J Med (2019) 381(1):36–46. doi: 10.1056/NEJMoa1814427 31269364

[23] Scolari F Delbarba E Santoro D Gesualdo L Pani A Dallera N. Rituximab or cyclophosphamide in the treatment of membranous nephropathy: the ri-cyclo randomized trial. J Am Soc Nephrol (2021). doi: 10.1681/ASN.2020071091 PMC8017548 33649098

[24] Dahan K Debiec H Plaisier E Cachanado M Rousseau A Wakselman L. Rituximab for severe membranous nephropathy: a 6-month trial with extended follow-up. J Am Soc Nephrol (2017) 28(1):348–58. doi: 10.1681/ASN.2016040449 PMC5198292 27352623

[25] Ruggenenti P Chiurchiu C Abbate M Perna A Cravedi P Bontempelli M. Rituximab for idiopathic membranous nephropathy: who can benefit? Clin J Am Soc Nephrol (2006) 1(4):738–48. doi: 10.2215/CJN.01080905 17699281

[26] Wang X Cui Z Zhang YM Qu Z Wang F Meng LQ. Rituximab for non-responsive idiopathic membranous nephropathy in a Chinese cohort. Nephrol Dial Transplant (2018) 33(9):1558–63. doi: 10.1093/ndt/gfx295 29149305

[27] Seitz-Polski B Dahan K Debiec H Rousseau A Andreani M Zaghrini C. High-dose rituximab and early remission in Pla2r1-related membranous nephropathy. Clin J Am Soc Nephrol (2019) 14(8):1173–82. doi: 10.2215/CJN.11791018 PMC6682825 31340979

[28] Moroni G Depetri F Del Vecchio L Gallelli B Raffiotta F Giglio E. Low-dose rituximab is poorly effective in patients with primary membranous nephropathy. Nephrol Dial Transplant (2017) 32(10):1691–6. doi: 10.1093/ndt/gfw251 27387472

[29] Hoxha E Thiele I Zahner G Panzer U Harendza S Stahl RA. Phospholipase A2 receptor autoantibodies and clinical outcome in patients with primary membranous nephropathy. J Am Soc Nephrol (2014) 25(6):1357–66. doi: 10.1681/ASN.2013040430 PMC4033365 24610926

[30] Fervenza FC Abraham RS Erickson SB Irazabal MV Eirin A Specks U. Rituximab therapy in idiopathic membranous nephropathy: a 2-year study. Clin J Am Soc Nephrol (2010) 5(12):2188–98. doi: 10.2215/CJN.05080610 PMC2994079 20705965

[31] Cravedi P Ruggenenti P Sghirlanzoni MC Remuzzi G. Titrating rituximab to circulating b cells to optimize lymphocytolytic therapy in idiopathic membranous nephropathy. Clin J Am Soc Nephrol (2007) 2(5):932–7. doi: 10.2215/CJN.01180307 17702725

[32] Courville J Nastoupil L Kaila N Kelton J Zhang J Alcasid A. Factors influencing infusion-related reactions following dosing of reference rituximab and pf-05280586, a rituximab biosimilar. BioDrugs (2021) 35(4):459–68. doi: 10.1007/s40259-021-00487-6 PMC8295162 34152584

[33] Fouda GE Bavbek S. Rituximab hypersensitivity: from clinical presentation to management. Front Pharmacol (2020) 11:572863. doi: 10.3389/fphar.2020.572863 33013416 PMC7508176

[34] Hamilton P Kanigicherla D Venning M Brenchley P Meads D. Rituximab versus the modified ponticelli regimen in the treatment of primary membranous nephropathy: a health economic model. Nephrol Dial Transplant (2018) 33(12):2145–55. doi: 10.1093/ndt/gfy049 29617884

[35] Cravedi P Remuzzi G Ruggenenti P. Rituximab in primary membranous nephropathy: first-line therapy, why not? Nephron Clin Pract (2014) 128(3-4):261–9. doi: 10.1159/000368589 25427622

